# Dual glycosylation of wall teichoic acid modulates the O‐antigen pattern and virulence in serovar 4b *Listeria monocytogenes*

**DOI:** 10.1002/mlf2.70041

**Published:** 2025-12-16

**Authors:** Hao Yao, Yuting Wang, Ruochen Wang, Zhengnan Dong, Zhenhua Wu, Luyong Wang, Yuelan Yin, Xin'an Jiao

**Affiliations:** ^1^ Jiangsu Key Laboratory of Zoonosis Yangzhou University Yangzhou China; ^2^ Key Laboratory of Prevention and Control of Biological Hazard Factors (Animal Origin) for Agrifood Safety and Quality, Ministry of Agriculture and Rural Affairs of the People's Republic of China Yangzhou University Yangzhou China; ^3^ Joint International Research Laboratory of Agriculture and Agri‐product Safety of the Ministry of Education Yangzhou China; ^4^ Jiangsu Co‐Innovation Center for Prevention and Control of Important Animal Infectious Disease and Zoonoses Yangzhou China

**Keywords:** ActA, galactosylation, glucosylation, *Listeria monocytogenes*, wall teichoic acid

## Abstract

Among the 14 serovars of *Listeria monocytogenes* (Lm), serovar 4b strains are the most predominant isolates linked to human listeriosis outbreaks‐a phenotype associated with their unique wall teichoic acid (WTA) decorated with galactose (Gal) and glucose (Glu). A wealth of knowledge is available for galactosylated‐WTA (Gal‐WTA) manipulating bacterial homeostasis and virulence, whereas the relationship between glucosylated‐WTA (Glu‐WTA) and Gal‐WTA in listerial physiology and pathogenesis remains unclear. Here, we find that Glu‐WTA and Gal‐WTA jointly constitute the O‐antigen pattern of serovar 4b Lm; however, Glu‐WTA specifically serves as the indispensable ligand for listeriophage LP4 adsorption. Moreover, the co‐operation between Glu‐ and Gal‐WTA increases biofilm formation and bacterial resistance to cationic antimicrobial peptide (CRAMP). We further demonstrate that Gal‐WTA modulates the anchoring of surface proteins, including IspC, Ami, and InlB. Additionally, dual glycosylated WTA interaction with ActA facilitates bacterial intracellular motility and dissemination. Consistently, Glu‐WTA significantly enhances bacterial colonization ability in the mesenteric lymph nodes (MLNs), ileum, liver, and brain of mouse, cooperating with Gal‐WTA to facilitate Lm dissemination to distant organs and tissues. In conclusion, we reveal the crucial roles of Glu‐WTA in synergizing with Gal‐WTA to modulate the integrity of the cell wall structure and exacerbate bacterial infection, providing a global understanding of the hypervirulence and pathogenicity of invasive serovar 4b Lm.

## Impact statement

Serovar 4b *Listeria monocytogenes* (Lm), which is decorated with galactosylated and glucosylated wall teichoic acid (WTA), accounts for the majority of clinical listeriosis cases. Here, we confirm that both galactosylated‐WTA (Gal‐WTA) and glucosylated‐WTA (Glu‐WTA) are the major O‐antigen determinants of serovar 4b Lm. Importantly, both Glu‐ and Gal‐WTA promote ActA aggregation with host actin. Additionally, Gal‐WTA modulates the surface association of glycine–tryptophan (GW) proteins, thus playing crucial roles in mediating bacterial proliferation and translocation. Moreover, the absence of either Glu‐ or Gal‐WTA remarkably attenuates the ability of bacteria to colonize the intestine and spread to deep organs. This finding highlights the crucial roles of Glu‐WTA in synergy with Gal‐WTA in the pathogenesis of serovar 4b Lm.

## INTRODUCTION

*Listeria monocytogenes* (Lm) is a major opportunistic Gram‐positive bacterium, causing severe listeriosis, including septicemia, meningitis, abortion, and neonatal infection in susceptible individuals^1^. Lm is a ubiquitous foodborne pathogen that consists of 14 serovars (SVs). Notably, SVs 4b, 1/2b, and 1/2a isolates account for 98% of clinical cases, of which 4b isolates from lineage I are associated with 50% of human listeriosis, in particular central nervous system (CNS) and maternal–neonatal (MN) listeriosis^2^, ^3^. SV 4b strains shared some common crucial virulence factors with other SV Lm, such as the two major internalins, InlA and InlB, the pore‐forming toxin listeriolysin O (LLO), and the actin polymerization factor ActA^4^, ^5^. In addition, SV 4b strains carry additional unique virulence systems, including island 3 (LIPI‐3) and LIPI‐4, which further enhance their pathogenicity in the host^6^. Moreover, *Listeria* adhesion protein (LAP) destabilizes tight junction proteins to promote crossing of SV 4b strains over the intestinal epithelial barrier and avoid the innate immune defense system as early as 12 h post gavage^7^, ^8^, ^9^. Although extensive research has been conducted on SV 4b Lm pathogenesis, only a few studies have explored the relationship between SVs and virulence. In *Listeria*, SVs comprise somatic (O) antigen and flagellar (H) antigen, with O‐antigen differentiation depending on the carbohydrate diversity of wall teichoic acid (WTA) in the cell wall. Moreover, gene clusters responsible for WTA biosynthesis have been demonstrated to make a key contribution to Lm infection^10^, ^11^. WTA is the most abundant carbohydrate on the surface of Gram‐positive bacteria. It is covalently attached to the peptidoglycan and extends outward^12^, ^13^, ^14^. In *Listeria*, WTA diversity is mainly determined by the glycosylation on the main chain polymer, which contains 20 to 30 repeating units^14^. Type I WTA (including SVs 1/2, 3, and 7) is decorated with l‐rhamnose (Rha) and/or *N*‐acetylglucosamine (GlcNAc), but the main chain of type II WTA (including SVs 4, 5, and 6) integrated *N*‐GlcNAc further modified with galactose (Gal) and/or glucose (Glu)^14^, ^15^. Previous studies have shown that *rmlTACBD* and *lmo1079* are involved in Rha and *N*‐GlcNAc modification in type I WTA, respectively^16^, ^17^. In addition, *gltA*, *gltB*, and *gtcA* are associated with glucosylation, and *gtcA*, *gttA*, *gttB*, and *galT* are responsible for galactosylation in type II WTA^10^, ^11^, ^18^, ^19^, ^20^, ^21^. Notably, SV 4b strains show the most complex type II WTA structure compared to SVs 4a, 4c, 4d, 4e, and 4h strains, characterized by the co‐modification of Gal and Glu on GlcNAc. Moreover, SV 4b isolates are more frequently associated with outbreak of listeriosis than SVs 1/2b, 1/2a, and 1/2c Lm strains. Deciphering the roles of Gal and Glu decoration of WTA is crucial for understanding the pathogenesis of SV 4b strains.

WTA is strongly involved in a variety of bacterial physiology processes, such as maintaining cell wall structure, protecting against antimicrobial peptides (AMPs), regulating bacterial homeostasis, and promoting virulence^12^, ^15^. In *Listeria*, l‐rhamnosylated WTA (Rha‐WTA) of SV 1/2a EGD‐e promotes surface anchoring of virulence proteins InlB and Ami, thereby regulating bacterial physiology and pathogenesis^16^, ^22^. Similarly, galactosylated‐WTA (Gal‐WTA) of SV 4b WSLC1042 interacts with the invasion factor InlB, enhancing bacterial virulence in a mouse infection model^10^. Consistently, we have previously reported that Gal‐WTA of SV 4h XYSN plays essential roles in anchoring of glycine‐tryptophan (GW) domain proteins, and contributing to bacterial virulence^11^, ^20^. Another study showed that WTA glucosylation in Lm is involved in the formation of SV‐specific surface antigens in SV 4b strains 4b1^18^. Meanwhile, glucosylated‐WTA (Glu‐WTA) is not required for the virulence of SV 4b strains 4b1 in A/J mice^23^. However, there is not enough evidence to elucidate whether Glu‐WTA alone or in synergy with Gal‐WTA plays critical roles in the physiological processes and pathogenesis of SV 4b Lm.

In this study, using the highly invasive SV 4b strain NTSN, an ovine‐derived isolate from a listeriosis outbreak, we demonstrated that Glu‐WTA contributes to the composition of O‐antigen, which can be specifically recognized by bacteriophage LP4. Additionally, Glu‐WTA cooperates with Gal‐WTA to regulate the bacterial biofilm formation and resistance to AMPs. Notably, in addition to Gal‐WTA, Glu‐WTA also influences the ability of the virulence protein ActA to mediate actin tail formation, thereby promoting bacterial intracellular multiplication and colonization in host organs. Our findings provide important insights into the pathogenesis of SV 4b strains through their unique disaccharide‐modified WTA.

## RESULTS

### Glu‐ and Gal‐WTA defines the O‐antigen in serovar 4b *Listeria*

**Figure 1:**
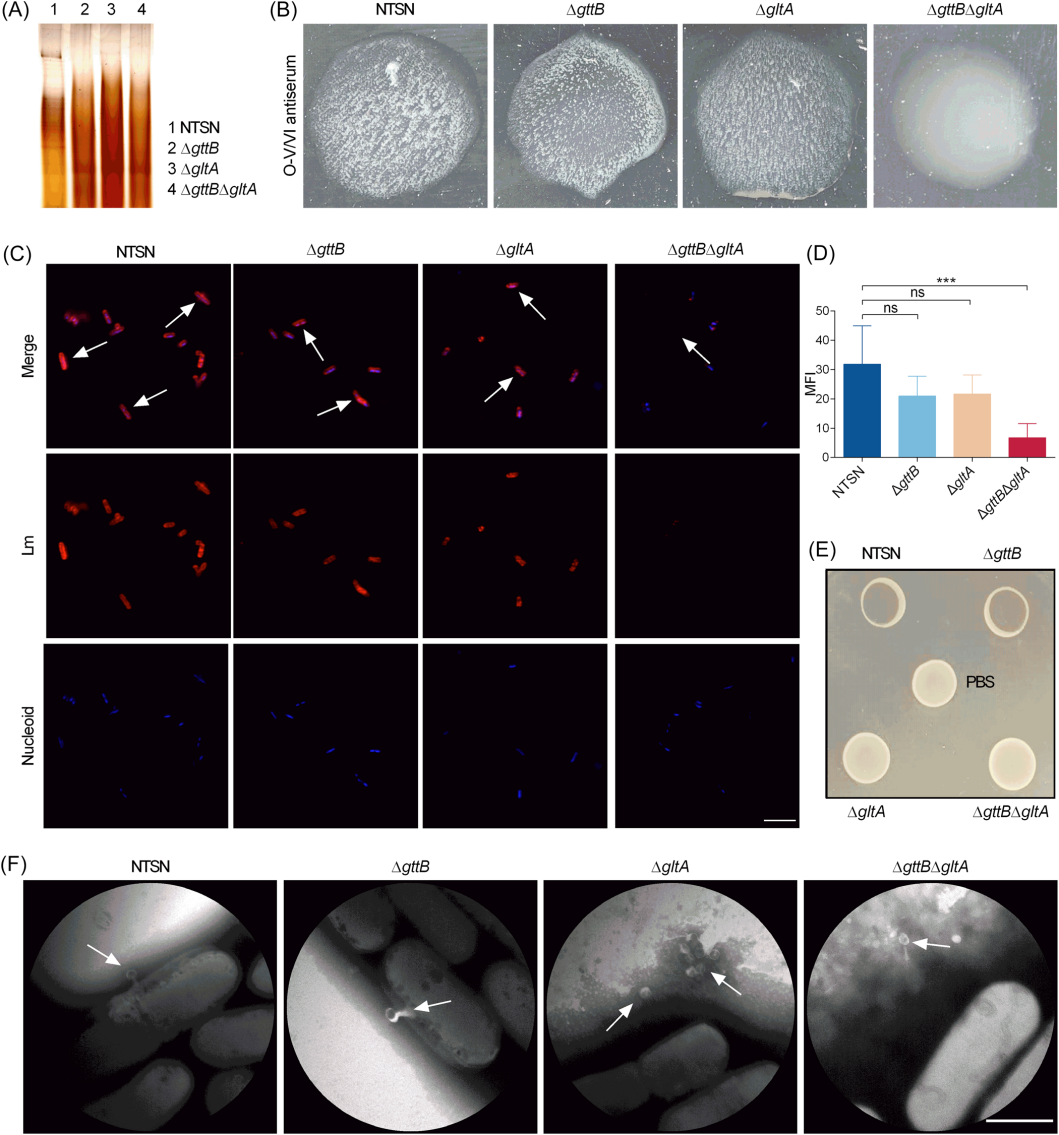
Wall teichoic acid (WTA) decorated with glucose (Glu) and galactose (Gal) maintains the structural integrity of cell wall. (A) Alcian blue‐stained polyacrylamide gel containing WTA extracted from wild‐type *Listeria monocytogenes* (Lm) NTSN, Δ*gttB*, Δ*gltA*, and Δ*gttB*Δ*gltA* mutants. (B) Images of agglutination patterns obtained from different Lm strains with O‐V/VI antiserum (Denka Seiken). (C) Confocal images of NTSN, Δ*gttB*, Δ*gltA*, and Δ*gttB*Δ*gltA* mutants. Bacteria were subsequently stained with O‐V/VI antiserum and Alexa Fluor 555‐conjugated rabbit antibody (red); the nucleoid was labeled with DAPI (blue). Magnification of all images: 3000×. Scale bar, 5 μm. (D) MFI on the surface of each *Listeria* analyzed using ImageJ software. The error bars represent SD. Statistical analyses were performed by Dunnett s multiple comparisons test. ***p < 0.001; ns, no significance. (E) Adsorption analysis of phage LP4 with NTSN, Δ*gttB*, Δ*gltA*, and Δ*gttB*Δ*gltA* strains. (F) Transmission electron microscopy analysis of the binding between bacteriophage LP4 and different *Listeria*. The white arrows indicate the locations of the listeriaphages. Magnification: 80,000×. Scale bar, 500 nm. MFI, mean fluorescence intensity.

The structural integrity of WTA is fundamental to *Listeria* O‐antigens. In this study, we tested whether Gal or Glu modification on WTA is associated with the structure of O‐antigen in SV 4b strains. According to the results of genome comparative analysis, LMntsn_1086, which consists of 700 amino acids, is 100% identical to GttB in SV 4b Lm WSLC1042^21^, and LMntsn_2713 is 100% identical to GltA in SV 4b Lm 4b1^18^. Then, we first constructed isogenic mutants deficient in Gal‐WTA (Δ*gttB*), Glu‐WTA (Δ*gltA*), and in both glycosylations (Δ*gttB*Δ*gltA*). Native polyacrylamide gel electrophoresis (PAGE) analysis showed that the WTA from the Δ*gttB*, Δ*gltA*, and Δ*gttB*Δ*gltA* mutants showed a distinct migration pattern compared to that of the wild‐type (WT) strain, suggesting that both *gttB* and *gltA* are associated with WTA modifications (Figure 1A).

To analyze whether both Gal‐WTA and Glu‐WTA are involved in the *Listeria* O‐antigens, slide agglutination assays were performed. The results showed that the WT NTSN strain, Δ*gttB* and Δ*gltA* mutants specifically reacted with O‐V/VI antiserum (Denka Seiken), while only the Δ*gttB*Δ*gltA* mutant lacked agglutination within less than 2 min (Figure 1B). Furthermore, the NTSN strain and the Δ*gltA* mutant could agglutinate with serogroup 4‐specific antiserum (Becton Dickinson), while the Δ*gttB* and Δ*gttB*Δ*gltA* mutants failed to agglutinate within 2 min (Figure S1A). Additionally, the NTSN, Δ*gttB*, and Δ*gltA* mutants were stained with fluorescein‐labeled O‐V/VI antiserum as well as *Listeria* O‐antiserum 4. The absence of both Glu‐WTA and Gal‐WTA prevented both antisera from binding to the double Δ*gttB*Δ*gltA* mutant (Figures 1C,D and S1B). Notably, surface fluorescence levels were significantly reduced on the Δ*gttB*Δ*gltA* mutant compared to the Δ*gttB* mutant using *Listeria* O‐antiserum 4 (Figure S1C). Collectively, these data indicated that both Glu and Gal modifications of type II WTA contribute to O‐antigen composition, conferring the *Listeria* SV 4b phenotype.

The structure of WTA is not only a major O‐antigen determinant but also a binding ligand for bacteriophage recognition in *Listeria* ^13^. Listerial phage LP4 was isolated from sewage samples in Jiangsu, China. The genome of phage LP4 is 139,306 bp in length, with a G + C content of 36.4%, and encodes 193 putative open reading frames. In this study, we found that phage LP4 specifically infected NTSN and the Δ*gttB* mutant, while the Δ*gltA* and Δ*gttB*Δ*gltA* mutants survived (Figure 1E). Moreover, transmission electron microscopy also showed that phage LP4 effectively bound to bacterial surfaces of NTSN and the Δ*gttB* mutant but failed to bind to the Δ*gltA* and Δ*gttB*Δ*gltA* mutants (Figure 1F), further demonstrating that glucosylation is an important component of the bacterial cell wall and may serve as a unique binding site for phage LP4 endolysins.

### Glu‐ and Gal‐WTA contributes to listerial homeostasis

**Figure 2:**
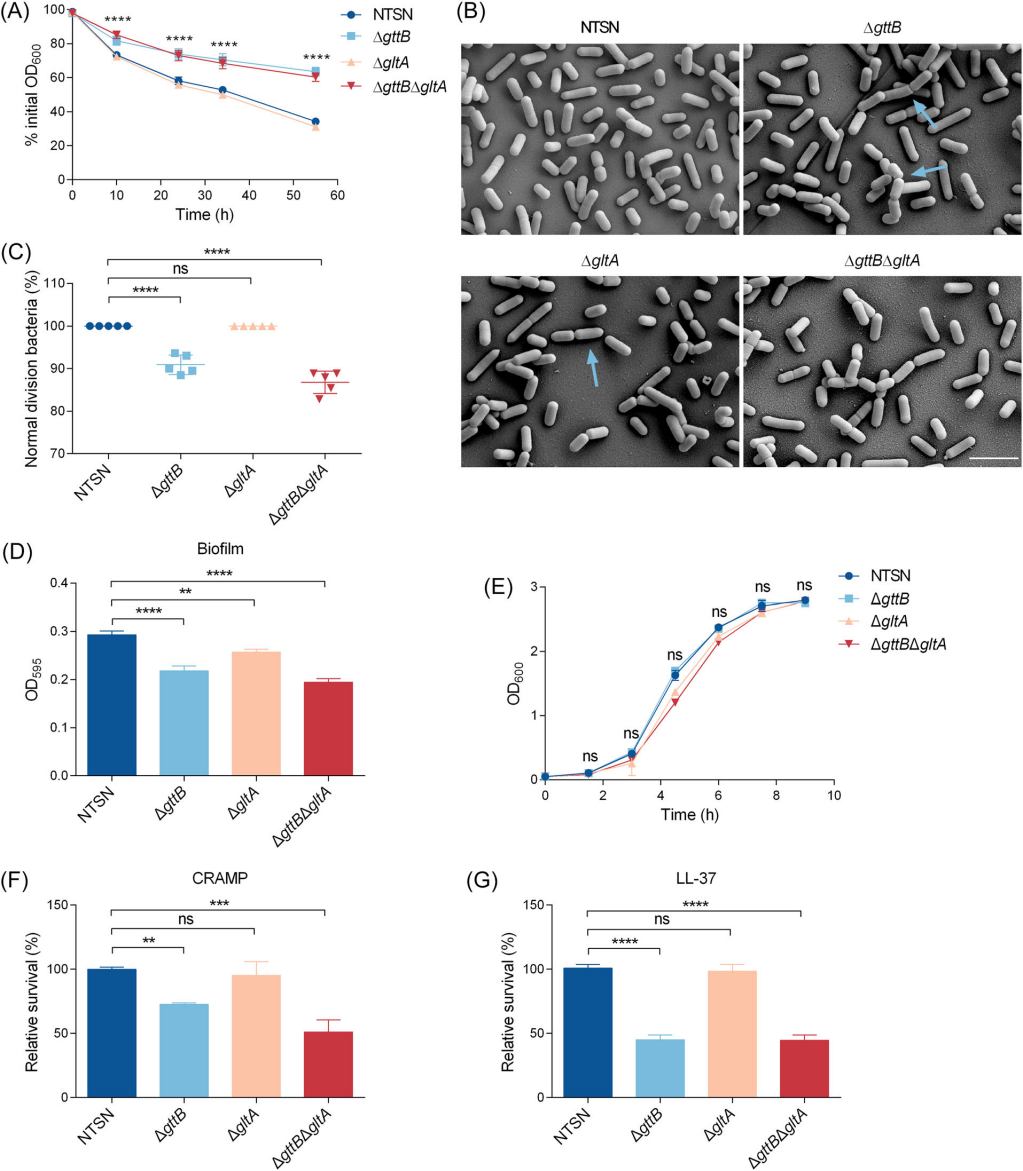
WTA decorated with Glu and Gal regulates listerial homeostasis. (A) The autolysin activity of NTSN, Δ*gttB*, Δ*gltA*, and Δ*gttB*Δ*gltA* strains. Listerial cells were washed and resuspended in 50 mM glycine‐HCl buffer (pH = 8.0). The initial OD_600_ values were adjusted to about 1.0 and monitored until clarification. All the error bars represented SD; *n* = 3 independent experiments. Statistical analyses were performed by Tukey's multiple comparisons test. ****p < 0.0001 (B) Scanning electron microscopy images depicting the division of NTSN, Δ*gttB*, Δ*gltA*, and Δ*gttB*Δ*gltA* cells. Magnification: 10,000×. Scar bar, 2 μm. (C) The number of abnormally dividing bacteria counted as the percentage of the total number of bacteria in five fields. (D) The contribution of Gal‐WTA and Glu‐WTA to biofilm formation. Quantification of overnight bacterial cultures was added to a sterile 96‐well plate and incubated for 48 h at 37°C. The levels (OD_595_) of the crystal violet present in destaining solution were measured. (E) Growing curves of NTSN, Δ*gttB*, Δ*gltA*, and Δ*gttB*Δ*gltA* strains measured in brain–heart infusion (BHI). (F, G) Roles of WTA‐glycosylation in the protection of Lm toward AMPs. Quantification of viable bacteria following treatment with Leucine‐Leucine 37 (LL‐37) (10 µg/ml) and cationic antimicrobial peptide (CRAMP) (15 µg/ml) at 37°C on growing cultures. All the error bars represented SD; *n* = 3 independent experiments. Statistical analyses were performed by Dunnett's multiple comparisons test. **p < 0.01; ***p < 0.001; ****p < 0.0001; ns, no significance.

We aimed to further investigate whether disaccharide‐modified WTA plays a key role in various physiological activities of bacteria. We first monitored the autolysin activity of NTSN, Δ*gttB*, Δ*gltA*, and Δ*gttB*Δ*gltA*. Based on OD_600_ values, the Δ*gttB* mutant showed significant deficiency in lysis compared to the WT strain. This phenotype was also observed in the mutants lacking both Gal and Glu (Δ*gttB*Δ*gltA*). However, there was no difference in autolytic behavior between the Δ*gltA* mutant and the WT strain (Figure 2A). Furthermore, we analyzed bacterial cell division ability by scanning electron microscopy. The absence of Gal affected normal bacterial division, with 12.9% of cells in the Δ*gttB* mutant and 13.5% in the Δ*gttB*Δ*gltA* mutant failing to separate effectively during cell division, resulting in the formation of elongated chains. However, the Δ*gltA* mutant, lacking Glu‐WTA, showed normal division similar to the WT strain (Figure 2B,C). These results demonstrated that Gal‐WTA, rather than Glu‐WTA, is essential for bacterial autolysis and cell division.

Polysaccharides are crucial components of bacterial biofilms, performing multiple functions, including maintaining biofilm structure and protecting cells from antimicrobials. In this study, we analyzed the impact of WTA glycosylation on biofilm formation in Lm SV 4b. Compared to the WT strain, the Δ*gttB* and Δ*gltA* mutants showed a significant reduction in biofilm formation. Moreover, the Δ*gttB*Δ*gltA* mutant showed an even greater reduction in biofilm‐forming ability (Figure 2D). This difference was independent of bacterial growth, as indicated by growth curve data (Figure 2E). Then, we further tested the role of WTA glycosylation in SV 4b Lm resistance to AMPs. After 2 h of incubation with CRAMP (15 µg/ml) and LL‐37 (10 µg/ml), the Δ*gttB* and Δ*gttB*Δ*gltA* mutants showed markedly increased susceptibility to AMPs compared to the WT strain, whereas there were no obvious survival defects in the Δ*gltA* mutant (Figure 2F,G). However, the differences in susceptibility to AMPs were more pronounced in the Δ*gttB*Δ*gltA* mutant in the presence of CRAMP, compared to the Δ*gttB* mutant (Figure 2F). These data suggested that Glu‐WTA synergizes with Gal‐WTA to promote Lm survival within and outside the host.

### Gal‐WTA is crucial for the surface association of GW proteins

**Figure 3:**
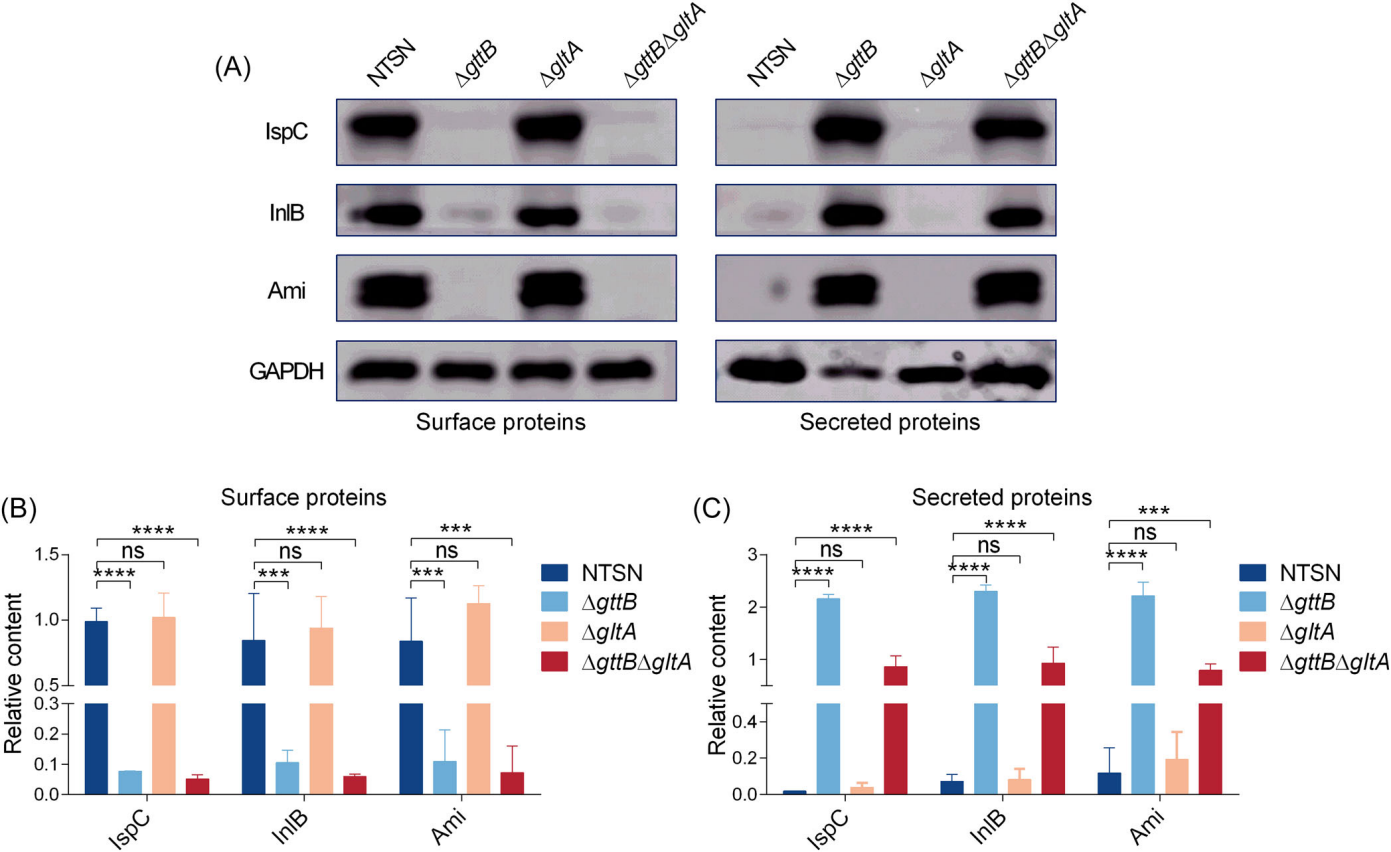
Gal‐WTA promotes the surface association of virulence proteins. (A) Western blot analysis of surface and secreted virulence proteins obtained from NTSN, Δ*gttB*, Δ*gltA*, and Δ*gttB*Δ*gltA* strains. The levels of GAPDH were used as a loading control. Proteins were detected using mouse polyclonal anti‐LygA serum, mouse polyclonal anti‐InlB serum, rabbit polyclonal anti‐Ami antiserum, and mouse polyclonal anti‐GAPDH serum. Protein levels were normalized using the BCA Protein Assay Kit (Beyotime). (B, C) Relative content of virulence proteins to GAPDH in surface proteins (B) and secreted proteins (C). The grayscale value of each protein in the Western blot was analyzed using ImageJ software, and the relative content was calculated as the ratio of the target protein band to the GAPDH band. Error bars represent SD; *n* = 3 independent experiments. Statistical analyses were performed by Tukey s multiple comparisons test. ***p < 0.001; ****p < 0.0001; ns, no significance.

Rha‐WTA and Gal‐WTA are required for the surface association of the GW proteins InlB and Ami^10^, ^11^, ^22^. Given this, we further assessed the role of Glu‐WTA in retaining surface virulence factors using Western blot analysis. Consistently, the absence of Gal in WTA (Δ*gttB* and Δ*gttB*Δ*gltA*) led to the release of GW proteins InlB and Ami from the cell wall surface into the culture supernatants. In contrast, all of these virulence proteins displayed surface association or secretion patterns in the Δ*gltA* mutant similar to those in the WT strain (Figure 3A). Moreover, the surface retention of unique autolysin IspC in SV 4b depended on Gal‐WTA rather than Glu‐WTA (Figure 3A). Furthermore, grayscale analysis showed that the surface levels of GW proteins InlB, Ami, and IspC in Gal‐WTA deletion strains were significantly lower than those in NTSN, while their levels in the supernatants showed an opposite trend. Consistently, the abundance of these GW proteins in cell wall or supernatants did not differ between the Δ*gltA* mutant and the WT strain (Figure 3B,C). These data confirmed that Gal‐WTA, rather than Glu‐WTA, is essential for the surface association of GW proteins.

### Glu‐ and Gal‐WTA promotes ActA‐mediated listerial intracellular dissemination

**Figure 4:**
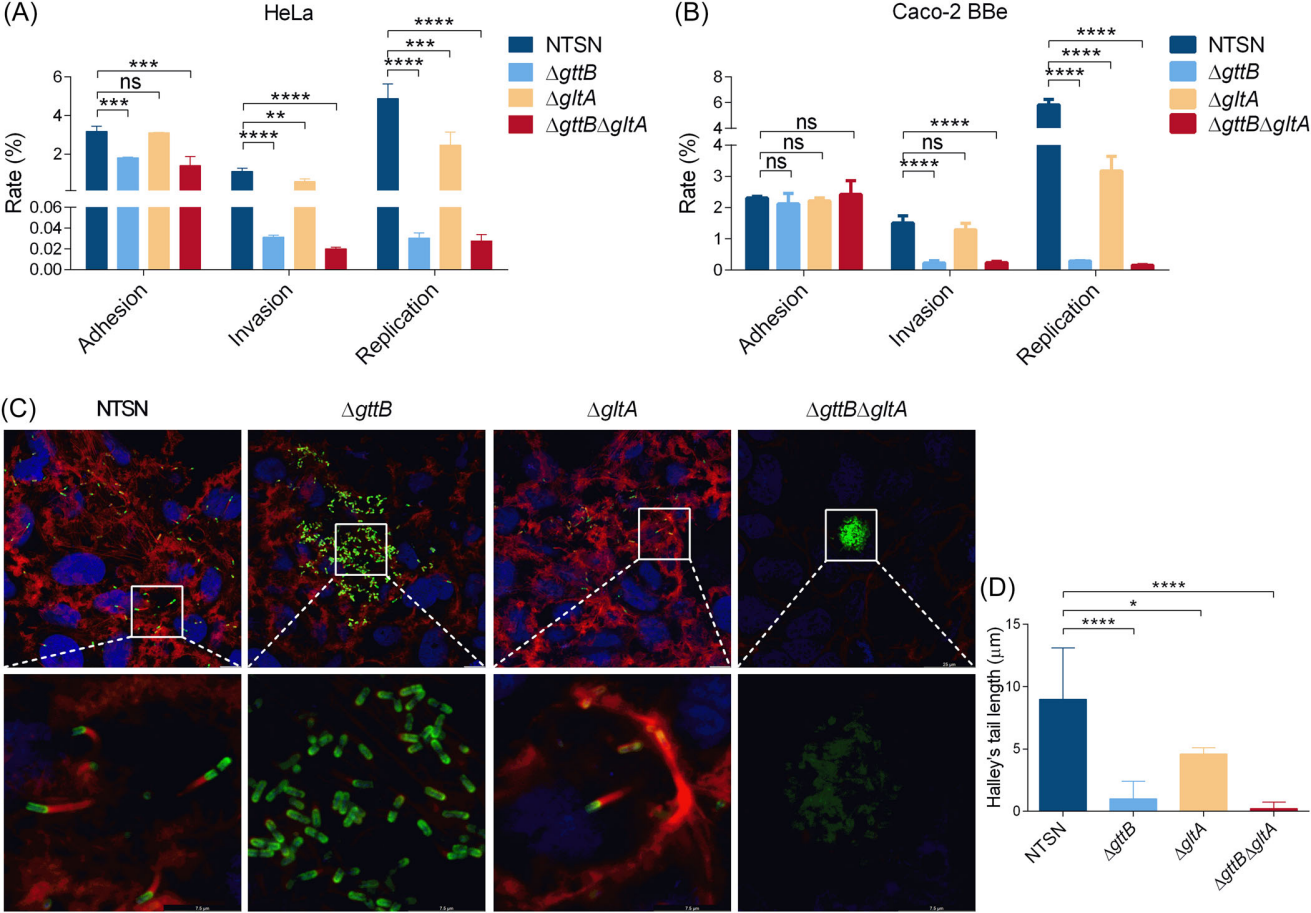
Glu‐WTA cooperates with Gal‐WTA to facilitate bacterial cellular invasiveness. (A, B) Quantitative analysis of adhesion, invasion, and replication of Lm in HeLa (A) and Caco‐2 BBe (B) cell lines. Cells were infected with NTSN, Δ*gttB*, Δ*gltA*, and Δ*gttB*Δ*gltA* strains at an MOI of 20 for 1 h and incubated for another 15 min or 2 h. The quantity of isogenic mutants was calculated relative to that of the WT strain (100%) according to the number of bacteria released from the cells. *n* = 3 independent experiments. (C) Confocal images of NTSN, Δ*gttB*, Δ*gltA*, and Δ*gttB*Δ*gltA* in Caco‐2 BBe cells at 7.5 hours post infection (hpi). ActA was visualized with an anti‐ActA monoclonal antibody (6F5) and goat Anti‐Mouse Alexa Fluor 488‐conjugated IgG (green), the actin cytoskeleton was labeled with phalloidin (red), and DNA was stained by DAPI (blue) following fixation and permeabilization of the samples. Magnification of all images: 1000× (up) and 3000× (down). (D) Average length of Halley's tail derived from five random fields. Error bars represent SD. Statistical analyses were performed by Dunnett s multiple comparisons test. *p < 0.05; **p < 0.01; ***p < 0.001; ****p < 0.0001; ns, no significance.

The ability of each deletion mutant to invade HeLa and Caco‐2 BBe cells was investigated. We observed that in HeLa cells, mutants lacking Gal‐WTA (Δ*gttB* and Δ*gttB*Δ*gltA*) showed impaired adhesion, invasion, and replication abilities compared to the WT strain. Meanwhile, the absence of Glu from WTA (Δ*gltA*) also significantly reduced bacterial invasion and replication abilities (Figure 4A). In addition, apart from slight differences in bacterial adherence to the cell surface, the single mutant Δ*gttB* and the double mutant Δ*gttB*Δ*gltA* showed markedly impaired invasion and replication abilities in Caco‐2 BBe cells. Moreover, the intracellular multiplication of the Δ*gltA* mutant was significantly lower than that of the WT strain (Figure 4B). These data suggested that Glu‐WTA and Gal‐WTA are major infection mediators, promoting the virulence of Lm.

ActA is the key determinant promoting listerial intracellular motility and cell‐to‐cell spread. Therefore, we analyzed the potential role of WTA glycosylation in the ability of the virulence protein ActA to aggregate host actin. The results showed that mutants lacking Gal‐WTA (Δ*gttB* and Δ*gttB*Δ*gltA*) failed to form actin tails, leading to impaired cell‐to‐cell spread. Although the Glu‐WTA‐deficient mutant (Δ*gltA*) retained intracytoplasmic motility, its average actin tail length was shorter than that of the parental strain (Figure 4C,D). These data indicated that Glu‐WTA and Gal‐WTA play crucial roles in ActA‐mediated intracellular motility, implying that specific WTA glycosylation promotes bacterial virulence.

### Glu‐WTA synergizes with Gal‐WTA to enhance the virulence of Lm

**Figure 5:**
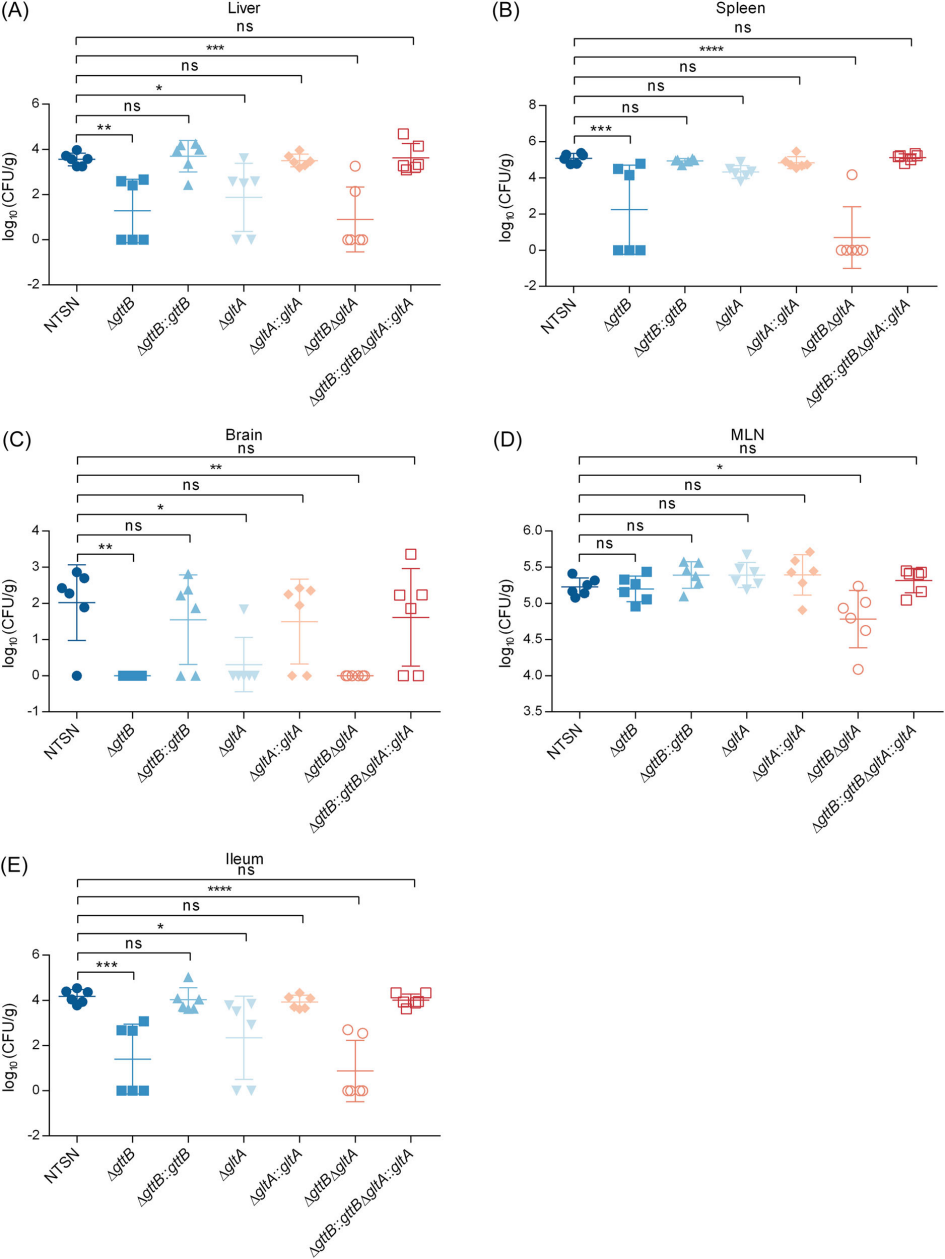
Dual glycosylation of WTA contributes to bacterial virulence. The colonization properties of the NTSN, Δ*gttB*, Δ*gttB*::*gttB*, Δ*gltA*, Δ*gltA*::*gltA*, Δ*gttB*Δ*gltA*, and Δ*gttB*::*gttB*Δ*gltA*::*gltA* strains in liver (A), spleen (B), brain (C), MLN (D), and ileum (E) were compared. Experiments were performed at 72 hpi. Each dot represents an organ from an infected mouse. log_10_ (CFUs/g) represents the mean of six mice per group. Error bars represent SD. Data were obtained from two independent experiments. *p < 0.05; **p < 0.01; ***p < 0.001; ****p < 0.0001; ns, no significance.

To analyze the roles of Gal‐ and Glu‐WTA in bacterial pathogenesis, we investigated the ability of each deletion mutant to colonize organs of C57BL/6 mice using an oral infection model. Compared to the WT strain, the Δ*gttB* and Δ*gltA* mutants showed significantly reduced colonization in the liver, ileum, and brain at 72 hours postinfection. In addition, the loss of Gal‐WTA markedly reduced bacterial colonization in the spleen. Notably, the double mutant Δ*gttB*Δ*gltA* showed significantly lower bacterial loads in the spleen, liver, mesenteric lymph nodes (MLNs), and ileum compared to either single mutant Δ*gttB*, Δ*gltA* or the WT strain NTSN. Moreover, complementation of the Δ*gttB*, Δ*gltA*, and Δ*gttB*Δ*gltA* mutants restored listerial colonization and its dissemination to distant organs and tissues (Figure 5A–E). These results indicated that WTA glycosylations contribute to the virulence of SV 4b Lm through both Gal‐WTA and Glu‐WTA.

## DISCUSSION

Wall teichoic acid (WTA) is a major anionic polymer ubiquitously found in the cell wall of Gram‐positive bacteria, playing crucial roles in maintaining cell wall integrity and function^12^. In *Listeria*, WTA shows structural diversity and is the major determinant of SV identity^24^. A previous study showed that the loss of galactosylation in WTA leads to a phenotype switch from SV 4b to SV 4d^10^. Similarly, galactose modification of WTA is essential for maintaining the integrity of O‐antigen in SV 4h XYSN^11^. These findings indicate that Gal‐WTA is a major component of O‐antigen in Lm. In addition to Gal‐WTA, GlcNAc residues on the type II WTA of SV 4b strains were further decorated with Glu^15^. In this study, we found that the absence of Gal on the WTA does not abolish the agglutination of NTSN with O‐V/VI antiserum, suggesting that Gal‐WTA alone is not the sole determinant of O‐antigens in SV 4b strains. Consistently, confocal microscopy images showed that the loss of disaccharide‐modified WTA resulted in the failure of O‐V/VI antiserum or O‐antiserum 4 to label the double mutant. These results further confirm that Glu‐WTA also contributes to the structural basis of O‐antigen and cooperates with Gal‐WTA to define the SV 4b of Lm. Moreover, a previous study has demonstrated that listeriophages enable the recognition of specific WTA structures, such as GlcNAc residues, O‐acetylated GlcNAc, rhamnose, and galactose^15^. In this study, we identified that Glu‐WTA may be a binding ligand of listeriophage LP4, highlighting its crucial roles in maintaining cell wall structure.

Teichoic acid (TA) represents the majority (~60%) of the total dry weight of bacterial cell walls, and plays crucial roles in regulating cell autolysis and division due to its complexity and heterogeneity^15^, ^25^. In *Bacillus subtilis*, TA modulates the abundance and activity of autolysin LytE, enhancing bacterial adaptability to diverse environments^26^. In *Staphylococcus aureus*, disruption of TA synthesis increases susceptibility to autolysis^27^. Moreover, the loss of Rha‐WTA in SV 1/2a and Gal‐WTA in SV 4h leads to a reduction of listerial autolytic activity^20^, ^22^. In this study, we also observed that Gal‐WTA‐deficient strains showed a significant decrease in autolytic activity, whereas the absence of Glu‐WTA did not induce this change, highlighting the important role of Gal‐WTA in bacterial autolysis. A previous study had confirmed that Gal‐WTA in SV 4h XYSN was involved in cell division^20^. Consistently, only the absence of Gal in WTA led to partial cell division defects in SV 4b strains. Taken together, these findings demonstrated that Glu‐WTA does not play a crucial role in regulating bacterial homeostasis.

AMPs interact with negatively charged WTA, facilitating their insertion into the bacterial cell membrane, thereby disrupting its integrity and inducing bacterial death^28^, ^29^. In *S. aureus*, d‐alanylation WTA reduces the electronegativity of the cell wall, enhancing bacterial sensitivity to AMPs^30^. WTA‐glycosylation also plays crucial roles in the resistance of Lm to AMPs. Previous studies demonstrated that the loss of Rha‐WTA in SV 1/2a leads to plasma membrane permeabilization and increased susceptibility to AMPs^16^. In addition, Gal‐WTA enhances bacterial tolerance to CRAMP and LL‐37 by maintaining cell wall integrity^11^. Based on these findings, listerial WTA‐glycosylation appears to be involved in maintaining the packing density and cross‐linking status of the bacterial cell wall, modulating AMPs' binding ability^31^. In this study, our results showed that the absence of Glu‐WTA had no effect on bacterial resistance to CRAMP, whereas concomitant deletion of both Gal‐ and Glu‐WTA significantly weakened the resistance to AMPs compared to a single Gal‐deficient strain. These results are consistent with previous findings showing that WTA‐glycosylation in Lm cooperates to enhance bacterial resistance to AMPs^32^. Biofilms, primarily composed of an extracellular polymeric substance (EPS) matrix and embedded planktonic cells, are critical for bacterial survival both within and outside the host^33^. Previous studies have shown that extracellular TA is the major polysaccharide component of the listerial biofilm matrix^34^. Furthermore, tunicamycin‐mediated inhibition of WTA biosynthesis reduced the bacterial ability to form biofilms^35^. Consistently, in this study, the deletion of either Glu‐WTA or Gal‐WTA in SV 4b NTSN significantly reduced the biofilm‐forming ability. Taken together, these results highlight the cooperative role of Glu‐WTA and Gal‐WTA in maintaining the cell wall integrity, protecting Lm against external environmental stressors.

Gram‐positive bacteria rely on WTA to provide anchoring sites for numerous virulence proteins, facilitating interactions with the diverse environments^36^, ^37^, ^38^. In Lm, defects in WTA synthesis resulted in significantly reduced surface levels of key virulence factors, such as InlB and LAP in Lm, while in *S. aureus*, similar defects diminish the presence of SasA^35^. In addition, the presence of Rha‐WTA in Lm SV 1/2a and Gal‐WTA in Lm SV 4b or 4h promotes the surface‐efficient association of virulence proteins InlB and Ami, thereby facilitating host infection^10^, ^20^, ^22^. However, a recent study reported that Glu‐WTA in SV 4b strain WSLC1042 is dispensable for the surface association of InlB and Ami^32^. Consistently, we also demonstrated that Glu‐WTA in SV 4b NTSN is not required for the surface anchoring of GW proteins. Moreover, we confirmed that IspC, another GW‐containing autolysin unique to Lm SV 4b, relies entirely on Gal‐WTA for its cell surface attachment. InlB interacts with c‐Met and gC1q‐R receptors to promote internalization of Lm in nonprofessional phagocytes, whereas InlA binding to E‐cadherin is required for listerial invasion on human epithelial cells, such as Caco‐2^39^. The loss of Gal on WTA significantly reduced surface anchoring of InlB, but did not affect the surface association of InlA^17^, leading to different phenotypes in the adhesion ability of the Δ*gttB*Δ*gltA* mutant to HeLa and Coco‐2 BBe cell lines. In addition, the role of Gal‐WTA in SV 4b NTSN is corroborated by previous findings showing that the absence of Gal‐WTA in Lm decreases the actin tail formation of ActA, leading to the loss of intracellular motility^11^, ^40^. However, our observations further revealed that Glu‐WTA is also responsible for bacterial cell‐to‐cell spread during infection. The deletion of Glu‐WTA led to shorter actin tails and partially reduced ability to escape from host cells. Altogether, these findings underscore the crucial roles of WTA diverse glycosylation patterns in surface anchoring of Lm virulence proteins. Meanwhile, how WTA glycosylation regulates ActA to aggregate host actin requires further exploration.

WTA plays an important role in the infection and pathogenesis of Gram‐positive bacteria. In *S. aureus*, high levels of WTA expression significantly promote skin abscess formation and increased virulence^41^. Similarly, the loss of Rha‐WTA or Gal‐WTA induced an obvious decrease in the virulence of Lm^10^, ^11^, ^16^. Our findings reveal that, in addition to Gla‐WTA, Glu‐WTA markedly enhances intracellular proliferation and promotes intestinal as well as systemic colonization of Lm in C57BL/6 mice following oral infection. This observation contrasts with earlier studies reporting no role for Glu‐WTA in the virulence of the SV 4b strain 4b1, a discrepancy likely attributable to differences in the mouse models or bacterial strain backgrounds employed^23^. Furthermore, our results demonstrate that Glu‐WTA cooperates with Gal‐WTA to facilitate bacterial infection. This synergistic effect may be explained by the ability of Glu‐WTA to increase Lm resistance to adverse conditions in the gut and limiting the capacity of ActA to induce actin polymerization. Notably, TA is the most abundant polysaccharide polymer on the surface of Gram‐positive bacteria, modulating host immune responses and bacterial virulence^42^. In *S. aureus*, α‐GlcNAc‐decorated WTA interacts with a nasal epithelial receptor SREC‐I in a charge‐dependent manner to promote bacterial infection^43^. Recent findings suggest that glycosylation of ribitol phosphate WTA (RboP‐WTA) with glucose compared to GlcNAc‐modified modification can impair immune recognition in *S. epidermidis* ^44^. Nevertheless, how WTA‐glycosylation in *Listeria* contributes to host‐pathogen interaction is poorly understood. Therefore, it is imperative to further explore the roles of Glu‐WTA as well as Gal‐WTA in immune recognition and response and fully elucidate Lm infection strategies.

In summary, this study highlights the crucial roles of Glu‐ and Gal‐WTA in SV 4b Lm physiological processes. Our findings indicate that Glu‐WTA synergizes with Gal‐WTA to maintain somatic antigen, enhancing Lm dissemination to distant organs and tissues. Moreover, disaccharide‐modified WTA is expected to become a crucial antimicrobial drug target, which could have significant clinical and public health implications for the prevention and control of listeriosis.

## MATERIALS AND METHODS

### Bacterial strains, cell lines, and animals

All the bacterial strains used in this study are listed in Table S1. Lm and *Escherichia coli* strains were cultured in brain–heart infusion (BHI; Becton Dickinson) and Luria‐Bertani (LB) media, respectively. The *Listeria* mutants were constructed using the thermosensitive vector pAULA plasmid as previously described, with the corresponding primers listed in Table S2, ^11^. Complementary strains Δ*gttB*::*gttB*, Δ*gltA*::*gltA*, and Δ*gttB*::*gttB*Δ*gltA*::*gltA* were generated based on the respective deletion mutants. *gttB* complementation was achieved by replacing the downstream CATATA sequence with CACATT, while *gltA* reversion was confirmed by substituting the tag sequence GGAGAG with GGCGAA following the termination codon. The human cervical carcinoma cell line HeLa and the human colorectal adenocarcinoma epithelial cell line Caco‐2 BBe were cultured at 37°C with 5% CO_2_ in Dulbecco's Modified Eagle Medium (DMEM, Gibco) supplemented with 10% fetal bovine serum (FBS, Gibco). Six‐week‐old female C57BL/6 mice were obtained from the Vital River Laboratory Animal Technology Co., Ltd.

### WTA PAGE analysis

The extraction and analysis of *Listeria* WTA were performed as previously described^20^. Specifically, the bacterial pellets were washed with buffer 1 (50 mM 2‐(N‐morpholino) ethanesulfonic acid (MES), pH 6.5), and then resuspended in buffer 2 (4% SDS, 50 mM MES, pH 6.5). The suspension was boiled in a 100°C water bath for 1 h, cooled, and centrifuged. The resulting pellet was subsequently washed with buffer 2, buffer 3 (2% NaCl, 50 mM MES, pH 6.5), and finally with buffer 1. The pellet was then resuspended in buffer 1 and disrupted using an ultrasonic homogenizer. Unbroken cells were removed by low‐speed centrifugation, followed by high‐speed centrifugation to collect the pellet. The pellet was resuspended in buffer 4 (20 mM Tris‐HCl, 0.5% SDS, and 20 µg/ml proteinase K, pH 8.0) and incubated at 50°C without agitation for 4 h. After incubation, the sample was centrifuged and the pellet was washed with buffer 3, followed by three washes with ddH_2_O to further remove residual SDS. To hydrolyze the WTA, the pellet was suspended in 0.1 M NaOH and incubated at room temperature (RT) with shaking for 16 h. Finally, the mixture was centrifuged to remove insoluble cell wall debris, and the supernatant containing WTA was neutralized with 1 M HCl.

The extracted WTA samples were mixed with nonreducing sample buffer and then subjected to native polyacrylamide gel electrophoresis (Native‐PAGE) at 4°C under a constant current of 20 mA, until the samples reached the bottom of the gel and electrophoresis was stopped. The gel was washed with ddH_2_O for 5 min and then incubated overnight in 200 ml of 0.1% Alcian blue staining solution (40% ethanol, 5% acetic acid). The next day, the gel was destained until no visible background staining remained. The gel was further silver‐stained using a Silver Staining Kit (Beyotime).

### Determination of O antigen

Fresh *Listeria* cells were mixed with O‐V/VI antiserum (Denka Seiken) or *Listeria* O‐antiserum 4 (Becton Dickinson) on glass slides. The presence or absence of agglutination was determined within 2 min under a light.

Log‐phase *Listeria* cultures were fixed with 4% paraformaldehyde at RT for 30 min, followed by blocking with PBS containing 5% BSA for 2 h. After washed with PBS, the bacteria were incubated with O‐V/VI antiserum (Denka Seiken, Japan) or *Listeria* O‐antiserum 4 (Becton Dickinson) at 37°C for 2 h. Subsequently, the bacteria were labeled at 37°C with Alexa Fluor 488‐ or 555‐conjugated goat anti‐rabbit antibodies (Abcam). For nucleoid staining, DAPI was added and incubated with the bacteria for 20 min. Finally, the bacteria were thoroughly washed with PBS, mounted on glass slides, and visualized using a Leica‐SP8ST‐WS confocal microscope with LAS X software.

### Bacteriophage adsorption analysis

Exponential‐phase bacteria (about 1 × 10^8^ CFU) were dropped into BHI plates. After 10 min, the phage LP4 was diluted with PBS and added at a 1:100 PFU/CFU ratio on the center of the bacteria. PBS was also added as a negative control. Then, the bacteriophage/listerial cell mixture was incubated overnight at 30°C to observe the plaque.

Log‐phase bacteria were incubated with phage LP4 for 5 min and then dropped onto a copper grid. After 15 min, excess liquid was removed with filter paper, followed by the addition of a drop of staining solution. The sample was left to stand for 1 min, excess stain was blotted off, and the grid was air‐dried. Finally, the sample was examined using a Hitachi HT7800 transmission electron microscope.

### Growth curve analysis

Overnight cultures of *Listeria* grown at 37°C with shaking at 180 rpm were collected, and the bacterial suspension was adjusted to an initial OD₆₀₀ of 0.05 using BHI medium. Then, 20 ml of the adjusted culture was transferred into Erlenmeyer flasks and further incubated under the same conditions. OD₆₀₀ readings were recorded every 1.5 h until the bacteria reached the stationary phase. For each strain, three parallel cultures were prepared. The growth curve was generated by plotting OD₆₀₀ values over time.

### AMPs susceptibility

The resistance of NTSN, Δ*gttB*, Δ*gltA*, and Δ*gttB*Δ*gltA* strains to antimicrobial peptides (AMPs) was assessed in vitro. Log‐phase bacteria were harvested and adjusted to an initial OD₆₀₀ of 0.8 (approximately 10⁹ CFU/ml) in PB medium (10 mM phosphate buffer, pH 7.4, supplemented with 1% BHI). The bacterial suspension was then diluted to 10⁴ CFU/ml. A volume of 100 µl of the diluted bacterial suspension was mixed with an equal volume of either 15 µg/ml CRAMP or 10 µg/ml LL‐37 in a 96‐well cell culture plate and incubated at 37°C for 2 h. After incubation, the mixtures were serially diluted in sterile PBS and plated on BHI agar plates for overnight incubation to determine the number of surviving bacteria. An equal volume of PBS without AMPs served as a negative control.

### Biofilm formation assay

The crystal violet staining method was used to assess the biofilm‐forming ability of Lm strains NTSN, Δ*gttB*, Δ*gltA*, and Δ*gttB*Δ*gltA*. Bacterial cultures were adjusted to an OD₆₀₀ of 0.1 using BHI medium. The adjusted suspensions were incubated statically at 37°C in test tubes for 48 h. After incubation, the supernatants were carefully removed, and the adherent biofilms were stained with 0.1% crystal violet solution for 15 min. The staining solution was then discarded, and the samples were washed with ddH_2_O. After drying at 55°C, the stained biofilms were solubilized with ethanol. Finally, OD₅₉₅ values were measured using a BioTek Synergy 2 microplate reader to evaluate biofilm formation.

### Bacteriolytic activity analysis

Autolysis assays were performed as previously described^22^. Log‐phase *Listeria* cells (OD_600_ = 0.8) were collected by centrifugation and washed twice with pre‐chilled distilled water. The cell pellets were then resuspended in glycine buffer (50 mM, pH 8.0) at an OD_600_ of 1.0. The OD_600_ was monitored at 30 min intervals during incubation at 37°C. Autolytic activity was assessed by calculating the ratio of the OD_600_ at each time point to the initial OD₆₀₀ value.

### Scanning electron microscopy

*Listeria* cells were cultured on glass slides until the exponential phase, and then washed and fixed with 2.5% glutaraldehyde at 4°C overnight. The samples were then rinsed three times with 0.1 M PBS and sequentially dehydrated in a graded ethanol series (30%, 50%, 70%, 80%, 90%, 95%, and 100%). After dehydration, samples were dried using a critical point dryer, coated with gold using an ion sputter coater, and examined under a GeminiSEM 300 scanning electron microscope.

### Western blot analysis

Surface and secreted proteins were extracted as previously described^11^. Protein concentrations were quantified using the BCA Protein Assay Kit (Beyotime) before Western blot analysis. Virulence proteins were detected using the following primary antibodies: mouse polyclonal anti‐InlB serum (prepared by our laboratory), mouse polyclonal anti‐LygA serum (prepared by our laboratory)^20^, rabbit polyclonal anti‐Ami antiserum^11^, and mouse monoclonal anti‐ActA antibody (6F5). GAPDH was used as a loading control and detected with mouse polyclonal anti‐GAPDH serum (prepared by our laboratory). Horseradish peroxidase (HRP)‐conjugated goat anti‐mouse IgG and goat anti‐rabbit IgG (both at 1:8000 dilution) were used as secondary antibodies. Blots were visualized through the Enhanced Chemiluminescence (ECL) Western blot analysis substrate.

### Fluorescence microscopy

Caco‐2 BBe cells were used to observe the cytosolic motility of Lm. At 7.5 hpi (MOI = 20:1), bacteria were sequentially labeled with mouse monoclonal antibody against ActA (6F5), followed by goat anti‐mouse IgG conjugated to Alexa Fluor® 488 (Abcam). The host cell cytoskeleton was marked with the buffer containing Phalloidin‐iFluor 555 (Abcam), and nuclei were counterstained with DAPI. Samples were imaged using a Leica‐SP8ST‐WS confocal microscope with LAS X software.

### In vitro adhesion, invasion, and replication assays

Cell infection assays were performed as previously described^11^. Bacteria from exponential‐phase cultures were pelleted and resuspended in DMEM to the appropriately concentration. Monolayers were incubated with Lm strains at a MOI of approximately 20 for 1 h at 37°C with 5% CO_2_. After infection, the cells were washed and lysed with 1 ml of 0.2% Triton X‐100 for 8 min. To determine the adhesion rate, lysates were serially diluted and plated on BHI agar to quantify intracellular bacteria. For evaluation of bacterial invasion and replication, cells were further incubated with DMEM containing 50 µg/ml gentamicin for 15 min and 2 h, respectively, to eliminate extracellular bacteria.

### Mice infections

Six‐week‐old female C57BL/6 mice (n = 6 per group) were used for in vivo infection experiments involving intragastric inoculation with the following Lm strains: NTSN, Δ*gttB*, Δ*gttB*::*gttB*, Δ*gltA*, Δ*gltA*::*gltA*, Δ*gttB*Δ*gltA*, and Δ*gttB*::*gttB*Δ*gltA*::*gltA* strains. Before infection, mice were starved for 16 h, with free access to water. Each mouse was orally administered approximately 1 × 10^9^ CFU of exponential‐phase bacteria suspended in a solution containing 30 mg/ml CaCO_3_. Bacterial counts in the spleen, liver, brain, MLN, and ileum were assessed on Day 3 postinoculation. A 1‐cm‐long ileum sample was collected, washed, and incubated in 30 ml of PBS supplemented with gentamicin (100 μg/ml) for 30 min^8^. Bacterial loads were determined by plating serial dilutions of homogenized tissue samples on BHI agar plates (spleen, liver, MLN, and brain samples) or CHROMagar plates (ileum).

### Data analysis

Statistical analyses were conducted using GraphPad Prism 6 (GraphPad Software, version 6.02). One‐way ANOVA, followed by Dunnett's multiple comparisons test, was used to compare group means relative to a control group. Two‐way analysis of variance with Tukey's multiple comparisons test was used for comparisons involving two or more groups. Statistical significance was defined as follows: *p < 0.05, **p < 0.01, ***p < 0.001, and ****p < 0.0001. Differences with *p*‐values > 0.05 were considered not to be statistically significant (ns).

## AUTHOR CONTRIBUTIONS

**Hao Yao**: Conceptualization; data curation; formal analysis; funding acquisition; investigation; methodology; validation; visualization; writing—original draft; writing—review and editing. **Yuting Wang**: Data curation; formal analysis; investigation; methodology; software; validation. **Ruochen Wang**: Data curation; formal analysis; investigation; methodology; software. **Zhengnan Dong**: Formal analysis; investigation; validation; visualization. **Zhenhua Wu**: Investigation; methodology. **Luyong Wang**: Data curation; resources. **Yuelan Yin**: Conceptualization; data curation; funding acquisition; methodology; project administration; resources; supervision; writing—original draft; writing—review and editing. **Xin'an Jiao**: Conceptualization; funding acquisition; methodology; project administration; resources; supervision; visualization; writing—review and editing.

## ETHICS STATEMENT

All animal experiments were conducted in accordance with the guidelines for the welfare and ethics of laboratory animals. Animals were housed in biosafety facilities following protocols approved by the Institutional Animal Ethics Committee of Yangzhou University (Approval No. 202106009).

## CONFLICT OF INTERESTS

The authors declare no conflict of interests.

## Supporting information

Supp_figure_1.

Supplementary_table_1.

Supplementary_table_2.

## Data Availability

All data that support the conclusions of this study are either included in the article or available from the corresponding author upon reasonable request.
