# Supplementary material for: Dual glycosylation of wall teichoic acid modulates the O‐antigen pattern and virulence in serovar 4b Listeria monocytogenes
Source: mLife. 2025 Dec 16;4(6):640–52. doi: 10.1002/mlf2.70041 (PMC12754626; doi:10.1002/mlf2.70041)

Supplementary figure 1

(A) Images of agglutination patterns obtained from Lm NTSN, the Δ*gttB*, Δ*gltA*, and Δ*gttB*Δ*gltA* mutants using *Listeria* O-antiserum 4 (Becton Dickinson, USA). (B) Confocal images of Lm NTSN, the Δ*gttB*, Δ*gltA*, and Δ*gttB*Δ*gltA* mutants. Bacteria were subsequently stained with *Listeria* O-antiserum 4 and Alexa Fluor 488-conjugated rabbit antibody (green), nucleoid was labeled with DAPI (blue). Magnification of all images: ×3000. Scale bars, 2 μm. (C) ImageJ software was used to analyze the fluorescence intensity on the surface of listerial cell. The error bars represented SD. ***$p < 0.001$, ****$p < 0.0001$, ns: no significance.

A

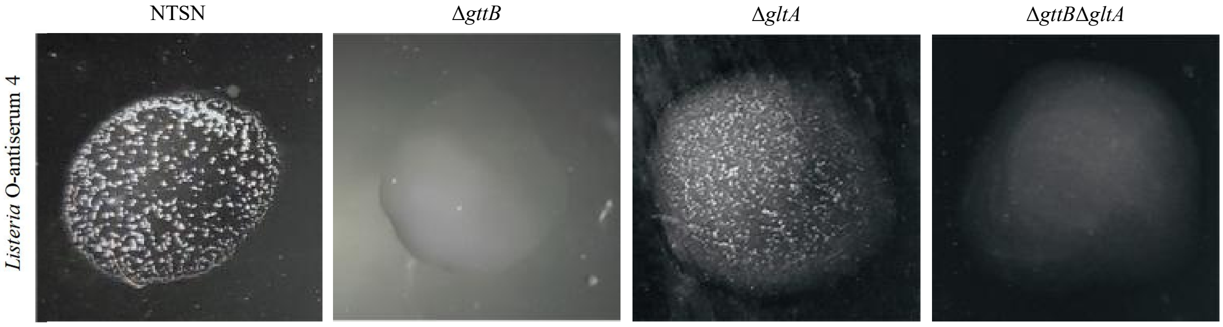

B

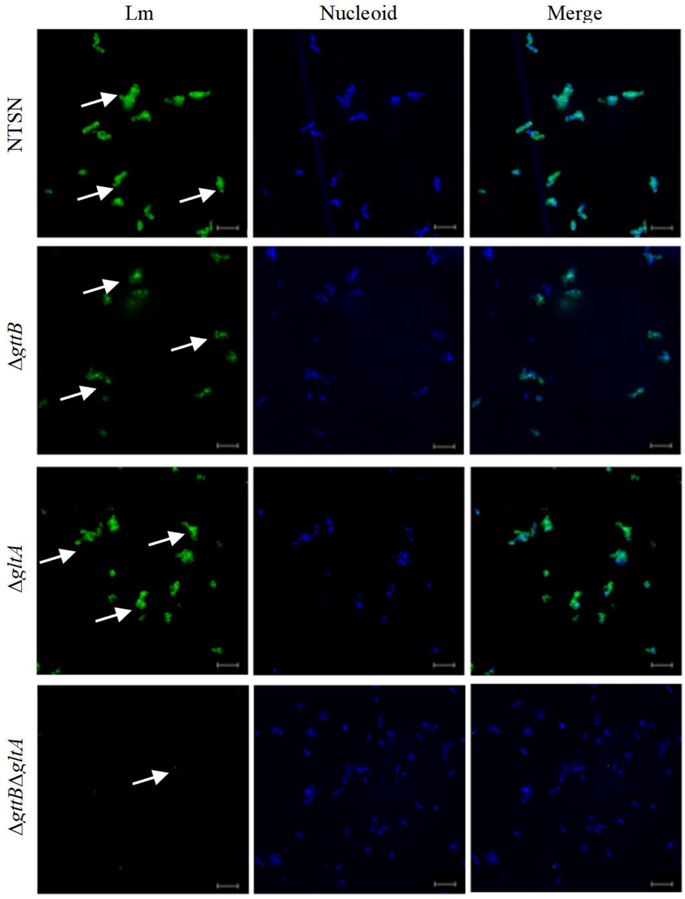

C

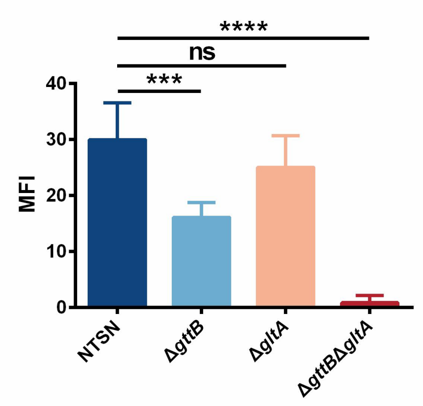

Supplement: Supplementary file 1 — Supp_figure_1. [file MLF2-4-640-s001.pdf]
