# Supplementary material for: Dual glycosylation of wall teichoic acid modulates the O‐antigen pattern and virulence in serovar 4b Listeria monocytogenes
Source: mLife. 2025 Dec 16;4(6):640–52. doi: 10.1002/mlf2.70041 (PMC12754626; doi:10.1002/mlf2.70041)
Supplement: Supplementary file 2 — Supplementary_table_1. [file MLF2-4-640-s002.docx]

**Supplementary table 1. Bacterial strains and plasmids used in this study**

| **Strains and plasmids** | **Description** | **Source** |
| --- | --- | --- |
| Lm NTSN | Wild type, serovar 4b | This lab |
| Δ*gttB* | *gttB* absent in Lm NTSN | This lab |
| Δ*gttB*::*gttB* | recombinant *gttB* in Δ*gttB* | This lab |
| Δ*gltA* | *gltA* absent in Lm NTSN | This lab |
| Δ*gltA*::*gltA* | recombinant *gltA* in Δ*gltA* | This lab |
| Δ*gttB*Δ*gltA* | *gttB* and *gltA* absent in Lm NTSN | This lab |
| Δ*gttB*::*gttB*Δ*gltA*::*gltA* | recombinant *gttB* and *gltA* in Δ*gttB*Δ*gltA* | This lab |
| *Escherichia coli* (DH5α) | Commercial strain used for cloning | This lab |
| pAULA | Em^R^ | Chakraborty, 1992 |
