# Supplementary material for: Dual glycosylation of wall teichoic acid modulates the O‐antigen pattern and virulence in serovar 4b Listeria monocytogenes
Source: mLife. 2025 Dec 16;4(6):640–52. doi: 10.1002/mlf2.70041 (PMC12754626; doi:10.1002/mlf2.70041)
Supplement: Supplementary file 3 — Supplementary_table_2. [file MLF2-4-640-s003.docx]

**Supplementary table 2. Primers used in this study**

| **Primers** | **Sequence (5’ to 3’)** |
| --- | --- |
| GttB-P1 | TTGTAAAACGACGGCCAGTGAATTCTTGATGCTGATTTACAAGATGAT |
| GttB-P2 | TGTCATCTATTATCTCCAGTTTTCCTTCTGCGTTGTTGCATGCCC |
| GttB-P3 | GGGCATGCAACAACGCAGAAGGAAAACTGGAGATAATAGATGACA |
| GttB-P4 | TGCATGCCTGCAGGTCGACTCTAGACTTACTTTCAATAGCCGCCCTTA |
| R-GttB-P1 | TGTAAAACGACGGCCAGTGAATTCTAGATTGAATACAAAAGGAGATGGTG |
| R-GttB-P2 | ATAGACTCTCCCTAATTCAAAAAATGTGTCATCTATTATCTCCAGTTTTCCTTC |
| R-GttB-P3 | GAAGGAAAACTGGAGATAATAGATGACACATTTTTTGAATTAGGGAGAGTCTAT |
| R-GttB-P4 | GCATGCCTGCAGGTCGACTCTAGAGTTTTCACTTTTCTTACTTTCAATA |
| GttB-W1 | GGATTTCGTTGTAGTATGCTTGG |
| GttB-W2 | AATTTGTTGGTTTTCACTTTTCTTA |
| GltA-P1 | TTGTAAAACGACGGCCAGTGAATTCGTCGGTCCAACTGGCTCT |
| GltA-P2 | GCACGGTGAAAATAAAACTCCATCCAAAATAAACTAAT |
| GltA-P3 | ATTAGTTTATTTTGGATGGAGTTTTATTTTCACCGTGC |
| GltA-P4 | TGCAYGCCTGCAGGTCGACTCTAGAGATGGCGACAAGTTAGGC |
| R-GltA-P1 | tgtaaaacgacggccagtgaattcGCAGCTTATGAGTATTGCGAGAAC |
| R-GltA-P2 | gaattcccattattTTCGCCTTAAAATTGATTTTCTTTG |
| R-GltA-P3 | ggcgaaAATAATGGGAATTCTAAATGAGAAAGTAGC |
| R-GltA-P4 | gcatgcctgcaggtcgactctagaGAGCTATCGTATCAAGGATTAATCCA |
| GltA-W1 | TTGATGTAAGTAAAGGTAAAATGGT |
| GltA-W2 | GCGGTCAGCAAGTAGGTGG |
